# The conundrum of postpartum thrombotic Microangiopathy: case report and considerations for management

**DOI:** 10.1186/s12882-019-1286-1

**Published:** 2019-03-14

**Authors:** Katharina Artinger, Gerald Hackl, Gernot Schilcher, Florian Eisner, Marion J. Pollheimer, Christoph Mache, Eva-Christine Weiss, Kathrin Eller, Philipp Eller

**Affiliations:** 1 Department of Internal Medicine, Clinical Division of Nephrology, Medical University of Graz, Graz, Austria; 2 Department of Internal Medicine, Intensive Care Unit, Medical University Graz, Auenbruggerplatz 15, A-8036 Graz, Austria; 3 Institute of Pathology, Medical University of Graz, Graz, Austria; 4 Department of Pediatrics and Adolescent Medicine, Division of General Pediatrics, Medical University of Graz, Graz, Austria; 5 Department of Obstetrics and Gynecology, Medical University of Graz, Graz, Austria

**Keywords:** Thrombotic microangiopathy, Preeclampsia, HELLP syndrome, Plasma exchange, Eculizumab

## Abstract

**Background:**

Microangiopathic hemolytic anemias and thrombocytopenias in pregnant or postpartum women constitute an interdisciplinary diagnostic and therapeutic challenge in the evaluation of thrombotic microangiopathies (TMA), where urgent care must be considered.

**Case presentation:**

We here report the case of a 21-year-old Somali woman, who was delivered by emergency caesarean section at 35 weeks of gestational age with acute dyspnea, placental abruption and gross edema due to severe preeclampsia/HELLP syndrome. After delivery, she developed acute kidney failure and thrombotic microangiopathy as revealed by kidney biopsy. The lack of early response to plasma exchange prompted extensive laboratory workup. Ultimately, the patient completely recovered with negative fluid balance and control of severe hypertension.

**Conclusions:**

This case report emphasizes the importance to differentiate between primary TMA syndromes and microangiopathic hemolytic anemias due to systemic disorders. Delayed recovery from preeclampsia/HELLP syndrome and malignant hypertension can clinically mimic primary TMA syndromes in the postpartum period.

**Electronic supplementary material:**

The online version of this article (10.1186/s12882-019-1286-1) contains supplementary material, which is available to authorized users.

## Background

Microangiopathic hemolytic anemias and thrombocytopenias in pregnant or postpartum women constitute an interdisciplinary diagnostic and therapeutic challenge in the evaluation of thrombotic microangiopathies (TMA), where urgent care must be considered for immediate treatment particularly of preeclampsia/HELLP (PE/HELLP) syndrome, thrombotic thrombocytopenic purpura (TTP), and complement-mediated thrombotic microangiopathy [1–3].

## Case presentation

**Fig. 1:**
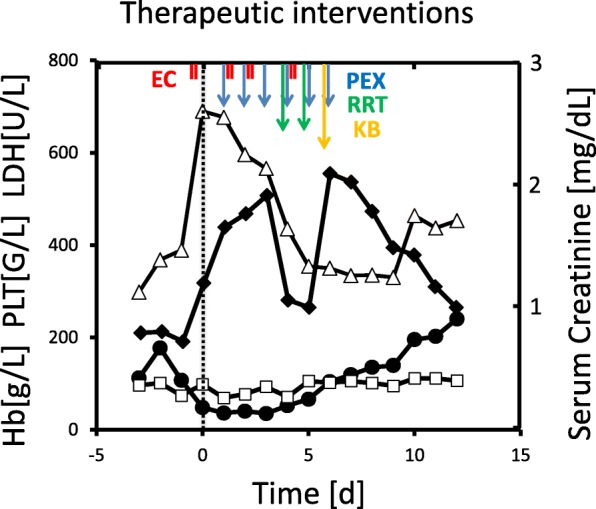
Time course showing laboratory parameters and therapeutic interventions: Platelet count (PLT, closed circles), serum lactate dehydrogenase (LDH, open triangles), hemoglobin (Hb, open squares) and serum creatinine (closed diamonds) are given on the ordinate as a function of time [days]. Day 0 is defined as the day of delivery (dotted black line). Red bars indicate infusion of erythrocyte concentrates (EC), blue arrows show treatment with plasma exchange (PEX), green arrows renal replacement therapy (RRT), and the orange arrows stands for kidney biopsy (KB), respectively

**Fig. 2:**
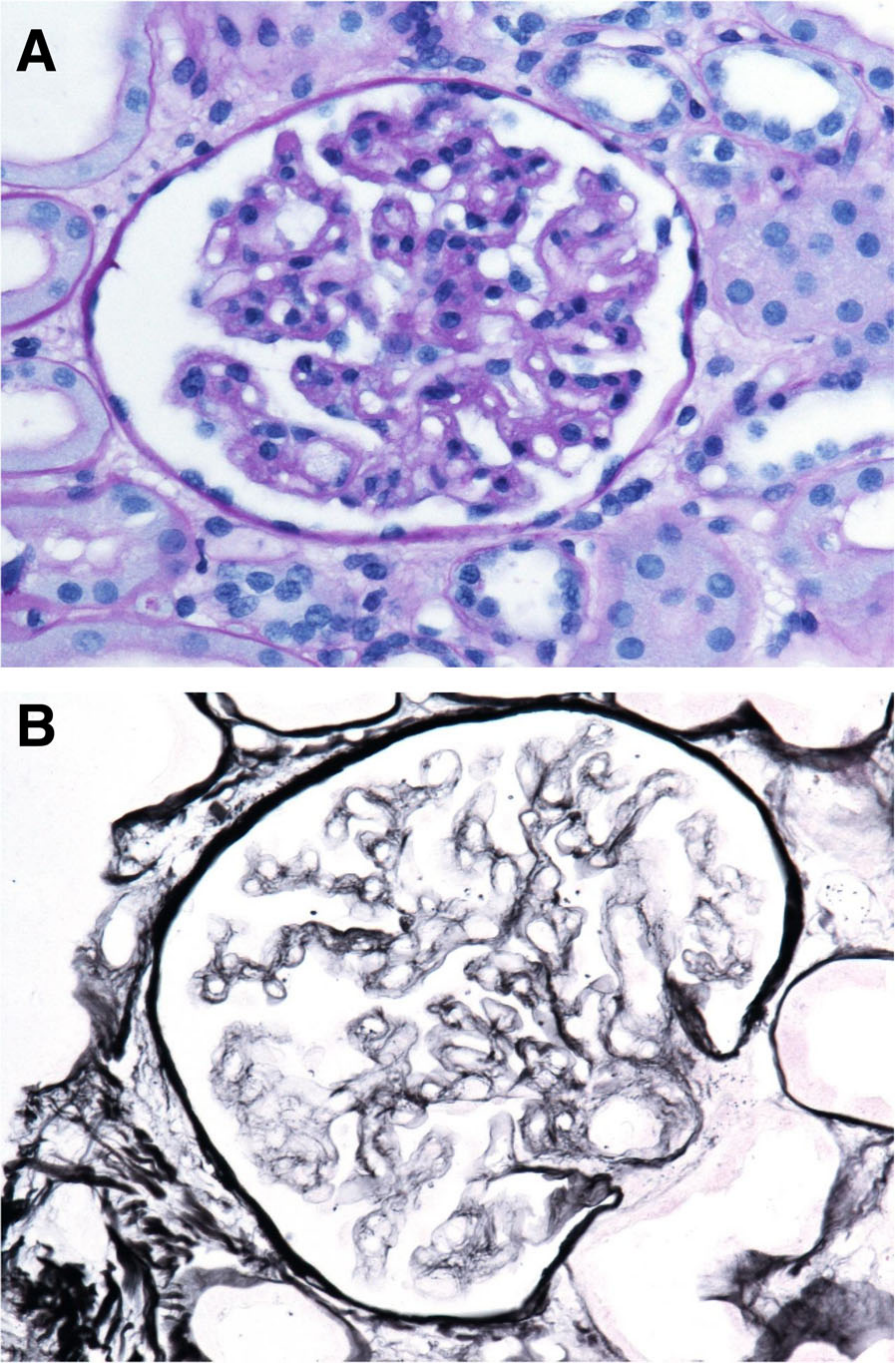
Representative renal biopsy pictures: **a** Periodic acid-Schiff reaction-(PAS) stained section showing glomerulopathy with thickened glomerular basement membranes, roundish capillary lumina and thrombotic obliteration of a capillary lumen. **b** Silver stained section illustrating segmental double contours of the capillary loops. Magnification × 400

**Fig. 3:**
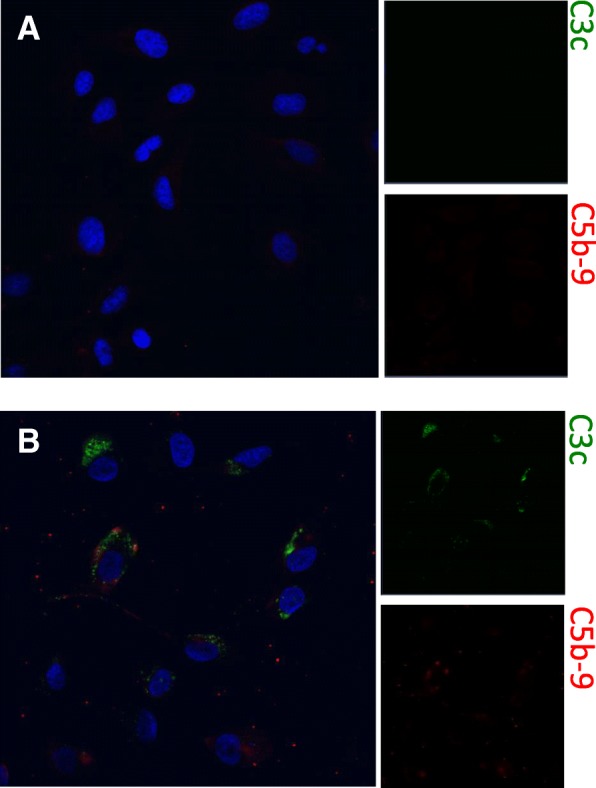
Complement deposition on endothelial cells: Endothelial cells were incubated with **a** serum from the index patient and **b** control serum from a patient with acute complement-mediated TMA due to complement factor H mutation. Serum of the index patient caused no deposition of C3c (FITC) and C5b-9 (Rhodamine) on non-activated endothelial cells. Magnification × 40

We here report the case of a 21-year-old Somali woman, who was delivered by emergency caesarean section at 35 weeks of gestational age with acute dyspnea, placental abruption and gross edema due to severe PE/HELLP syndrome. This was her first pregnancy, which had been uneventful up to the 34th gestational week. Her soluble fms-like tyrosine kinase-1/placental growth factor ratio 2 days prior was 211.4 [4]. After surgery, the patient was immediately transferred to Intensive Care Unit because of lung edema. The laboratory analysis revealed anemia of 7.4 g/dL, thrombocytopenia of 50 G/L, a negative coombs test, increased serum lactate dehydrogenase of 690 U/L, increased bilirubin of 2.2 mg/dL, elevated aspartate transaminase of 150 U/L, elevated alanine transaminase of 140, creatinine of 1.19 mg/dL, and no detectable haptoglobin levels (< 0.09 g/L). The peripheral blood smear showed manifold schistocytes (2.8%) and the activated prothrombin time was 38.2 s (Additional file 1: Table S1). The PLASMIC score was high indicating a high pretest probability for TTP (> 90%) [5]. The patient displayed elevated systolic blood pressure between 160 and 200 mmHg despite of intensive blood pressure lowering medication including urapidil, nifedipin, furosemide, and dihydralazine. As concern for the diagnosis TTP was strong, we immediately initiated plasma exchange therapy (PEX) and glucocorticoid medication, and proceeded with further diagnostic evaluation over the next days (Fig. 1). While undergoing PEX, the renal retention parameters slowly increased over the next 4 days, reaching a serum creatinine level of 2.09 mg/dL and an estimated glomerular filtration rate of 33 mL/min/1. 73m^2^. In parallel, fibrinogen levels decreased to a nadir of 103 mg/dL, and the thrombocyte count was still as low as 35 G/L on the 4th postoperative day. This decrease was associated with a peak of D-dimer level (26.27 mg/L) on the 6th postoperative day. The lack of early response to PEX prompted us to discuss the need for anti-complement therapy with eculizumab and to seek for other causes of the patient’s symptoms. There was no retained placental rest after delivery. In the meantime, ADAMTS13 activity had been measured and was found to be only slightly decreased to 39–63%, thus excluding the diagnosis of TTP [6]. Shiga-toxin, malaria parasites, and HIV antigen/antibodies were not detectable, and the hepatitis B and C serology tests were negative. The screening test for antibodies to extractable nuclear antigens (ENA), antinuclear, antiphospholipid and anticardiolipin antibodies, as well as serum C3c (0.917 g/L) and serum C4 levels (0.113 g/L) were within normal ranges. Urinary analyses revealed an albuminuria of 4.2 g/g and 40% acanthocytes, respectively. Since the patient presented with anasarca, somnolence, partial respiratory insufficiency due to lung edema and pleural effusions as well as still poorly controlled hypertension, we initiated a continuous renal replacement therapy with ultrafiltration on the 4th postoperative day, reducing the body weight of the patient from 70 kg to 49.5 kg in four days. Furthermore, PEX was daily continued. Along with the negative fluid balance of 20.5 L, the patient drastically improved both clinically and with the laboratory parameters. In parallel, kidney biopsy was performed on the 6th postoperative day, which revealed a residual thrombotic microangiopathy and signs of malignant hypertension such as doubling of the basal membrane as well as mild tubular necrosis. Immune complex nephritis, e.g. lupus nephritis, was excluded (Fig. 2). Therefore, we stopped PEX on the 6th day postpartum, after having reached a thrombocyte threshold of >100G/L. Dose and number of antihypertensive medication were drastically decreased. In a serum sample drawn prior to PEX, we found no evidence of complement activation using an in-vitro assay for complement deposition on non-activated endothelial cells (Fig. 3). C3c and C5b-9 complement deposition assays were performed as described previously by Noris et al. [7] Moreover, there were neither anti-complement factor H antibodies as determined by ELISA, nor mutations of atypical hemolytic uremic syndrome-related genes *ADAMTS13, C3, CFB, CFB, CFH, CFHR1, CFHR2, CFHR3, CFHR4, CFHR5, CFI, DGKE, MCP/CD46, MMACHC,* and *THBD* as detected by next-generation sequencing [8]. The patient completely recovered without further need for renal replacement therapy and/or PEX. Thrombocyte count increased to 240 G/L, and creatinine serum levels decreased to 0.99 mg/dL on the 12th day after delivery. The patient was discharged from Intensive Care Unit after 10 days, and dismissed from Hospital on the 16th day postpartum without any chronic impairment of glomerular filtration rate. Twenty-eight days after delivery, the glomerular filtration rate was 127.7 mL/min/1. 73m^2^, and serum creatinine was 0.64 mg/dL. Moreover, urinary albumin/creatinine ratio recovered from 4.2 g/g to 0.55 g/g within 4 weeks.

The patient had given birth to a daughter who had an Apgar score of 8/9/10, a body weight of 2260 g (26th percentile), and a length of 46 cm. The daughter did not have increased perinatal morbidity.

## Methods for cell culture and complement deposition assay

Ea.hy 926 cells (human endothelial cell line) were cultured in DMEM low Glucose medium (Gibco, Thermo Fisher Scientific, Waltham, MA, USA) containing 10% fetal bovine serum, Antibiotic-Antimycotic (Gibco, Thermo Fisher Scientific), and HAT Supplement (Gibco, Thermo Fisher Scientific) on cell chamber slides and used when confluent. C3c and C5b-9 complement deposition assays were performed as described previously. Briefly, cells were incubated with patient serum (1:2 dilution) or control serum for 4 hours. Cells were then stained with FITC-conjugated polyclonal rabbit anti-human C3c complement antibody (F0201, DAKO, Glosturp, Denmark) and monoclonal mouse anti-human C5b-9 antibody (ab66768, Abcam, Cambridge, MA, USA), followed by secondary antibody incubation with Rhodamine (TRITC)-conjugated goat anti-mouse (115–025-146, Jackson Immuno Research Laboratories, West Grove, PA, USA). Rb IgG Isotype Control FITC (PA5–23092, Thermo Scientific) and Mouse IgG2a Isotype Control (MCA929, Bio-Rad, Hercules, CA, USA) were used as appropriate isotype control antibodies. Stained cells were mounted with ProLong Gold Antifade Mountant with DAPI (Thermo Scientific). Evaluation of C3 and C5b-9 complement deposition on human endothelial cells was performed on a LSM 510 confocal microscope (Zeiss, Oberkochen, Germany).

## Discussion and conclusions

Taken together, this case report emphasizes the importance to differentiate between primary TMA syndromes and microangiopathic hemolytic anemias due to systemic disorders [3–5]. Delayed recovery from PE/HELLP syndrome and severe hypertension can clinically mimic primary TMA syndromes in the postpartum period. Since the diagnostic approach is costly in terms of time, it is nevertheless important to start standard therapy including PEX and revisit the first tentative diagnosis with the incoming diagnostic results as well as the clinical course of disease.

## Additional file

Additional file 1: **Table S1.** Normal ranges of laboratory parameters. (DOCX 16 kb)

## Abbreviations

PE/HELLP: Preeclampsia/HELLP syndrome
PEX: Plasma exchange therapy
TMA: Thrombotic microangiopathy
TTP: Thrombotic thrombocytopenic purpura
