# Supplementary material for: The conundrum of postpartum thrombotic Microangiopathy: case report and considerations for management
Source: BMC Nephrol. 2019 Mar 14;20:91. doi: 10.1186/s12882-019-1286-1 (PMC6417121; doi:10.1186/s12882-019-1286-1)
Supplement: Supplementary file 1 — Table S1. Normal ranges of laboratory parameters. (DOCX 16 kb) [file 12882_2019_1286_MOESM1_ESM.docx]

**Additional file 1: Table S1:** Normal ranges of laboratory parameters

|  | **Normal Ranges** |
| --- | --- |
| **Hemoglobin** | 12.0-15.3 g/dL |
| **Platelets** | 140-440 G/L |
| **Serum creatinine** | -1.0 mg/dL |
| **Serum bilirubin** | 0.1-1.2 mg/dL |
| **Alanine transaminase** | -35 U/L |
| **Aspartate transaminase** | -30 U/L |
| **Lactate dehydrogenase** | 120-240 U/L |
| **Haptoglobin** | 0.3-2.0 g/L |
| **Fibrinogen** | 210-400 mg/dL |
| **Actiavted prothrombin time** | 26-36 s |
| **D-dimer** | -0.5 mg/L |
| **Complement 3c** | 0.9-1.8 g/L |
| **Complement 4** | 0.1-0.4 g/L |
| **CH50** | 31.6-57.6 u/mL |
| **ADAMTS13** | 50-110 % |
